# SRPX2 Is a Novel Chondroitin Sulfate Proteoglycan That Is Overexpressed in Gastrointestinal Cancer

**DOI:** 10.1371/journal.pone.0027922

**Published:** 2012-01-05

**Authors:** Kaoru Tanaka, Tokuzo Arao, Daisuke Tamura, Keiichi Aomatsu, Kazuyuki Furuta, Kazuko Matsumoto, Hiroyasu Kaneda, Kanae Kudo, Yoshihiko Fujita, Hideharu Kimura, Kazuyoshi Yanagihara, Yasuhide Yamada, Isamu Okamoto, Kazuhiko Nakagawa, Kazuto Nishio

**Affiliations:** 1 Department of Genome Biology, Kinki University School of Medicine, Osaka-Sayama, Osaka, Japan; 2 Department of Medical Oncology, Kinki University School of Medicine, Osaka-Sayama, Osaka, Japan; 3 Department of Life Science, Faculty of Pharmacy, Yasuda Women's University, Asaminami-ku, Hiroshima, Japan; 4 Department of Medical Oncology, National Cancer Center Hospital, Chuo-ku, Tokyo, Japan; Technische Universität München, Germany

## Abstract

SRPX2 (Sushi repeat-containing protein, X-linked 2) has recently emerged as a multifunctional protein that is involved in seizure disorders, angiogenesis and cellular adhesion. Here, we analyzed this protein biochemically. SRPX2 protein was secreted with a highly posttranslational modification. Chondroitinase ABC treatment completely decreased the molecular mass of purified SRPX2 protein to its predicted size, whereas heparitinase, keratanase and hyaluroinidase did not. Secreted SRPX2 protein was also detected using an anti-chondroitin sulfate antibody. These results indicate that SRPX2 is a novel chondroitin sulfate proteoglycan (CSPG). Furthermore, a binding assay revealed that hepatocyte growth factor dose-dependently binds to SRPX2 protein, and a ligand-glycosaminoglycans interaction was speculated to be likely in proteoglycans. Regarding its molecular architecture, SRPX2 has sushi repeat modules similar to four other CSPGs/lecticans; however, the molecular architecture of SRPX2 seems to be quite different from that of the lecticans. Taken together, we found that SRPX2 is a novel CSPG that is overexpressed in gastrointestinal cancer cells. Our findings provide key glycobiological insight into SRPX2 in cancer cells and demonstrate that SRPX2 is a new member of the cancer-related proteoglycan family.

## Introduction

Sushi repeat protein X-linked 2 (SRPX2) was first identified as a gene up-regulated in pro-B leukemia cells and was described as sushi-repeat protein up-regulated in leukemia (SPRUL, [1]). Several years later, SRPX2 was found to be responsible for rolandic seizures associated with oral and speech dyspraxia and mental retardation [2]. The disease-causing mutation (N327S) and a second mutation (Y72S) of SRPX2 were identified, and these mutations resulted in the gain-of-N-glycosylated form of the mutant protein [2]. Although the molecular and biological functions of SRPX2 have been unknown for a long time, a recent study clearly demonstrated that SRPX2 binds to urokinase plasminogen activator receptor (uPAR) in a ligand/receptor interaction and that SRPX2 mutations led to an increase in the SRPX2/uPAR binding affinity [3]. In the vascular endothelial cells, Srpx2 regulates endothelial cell migration and tube formation, and the interaction of SRPX2 and uPAR is also involved in the early phases of endothelial remodeling during angiogenesis [4].

Recently, we demonstrated that SRPX2 is overexpressed in gastric cancer tissue and that expression was associated with a poor clinical outcome [5]. SRPX2 enhances cellular migration and adhesion in gastric cancer cells and, interestingly, the conditioned-medium obtained from SRPX2-producing cells increased the cellular migration activity and cellular adhesion [5]. We further examined SRPX2, focusing on a biochemical analysis in this study.

## Materials and Methods

### Cell culture

HEK293 was maintained in DMEM medium and SNU-16 and MKN7 were maintained in RPMI1640 medium supplemented with 10% FBS. HUVEC (human umbilical vein endothelial cells) was maintained in Humedia-EG2 (KURABO, Tokyo, Japan) medium with 1% FBS under the addition of EGF and FGF-2. The cells were maintained in a 5% CO_2_-humidified atmosphere at 37°C. These cell lines were obtained from the Japanese Collection of Research Bioresources Collection (Sennan-shi, Osaka).

### Western blotting analysis

The western blotting analysis has been previously described [6]. In belief, cell pellets were lysed in RIPA buffer (Tris-HCl: 50 mM, pH 7.4; NP-40: 1%; Na-deoxycholate: 0.25%; NaCl: 150 mM; EDTA: 1 mM; phenylmethyl-sulfonyl fluoride: 1 mM; aprotinin, leupeptin, pepstatin: 1 mg/ml each; Na3VO4: 1 mM; NaF: 1 mM). Cell extracts were electrophoresed on 7.5% (w/v) polyacrylamide gels and transferred to a polyvinylidene di-fluoride membrane (Nihon Millipore, Tokyo, Japan). The membrane was incubated in Tris-buffered saline containing 0.5% Tween 20 with 3% BSA and then reacted with the primary antibodies and the HRP-conjugated secondary antibody for 90 min each. Visualization was achieved with an enhanced chemiluminescent detection reagent (Amersham Biosciences, Buckinghamshire, UK). The following antibodies were used: anti-HA high affinity (Roche Applied Science, Mannheim, Germany), anti-SRPX2 [5] and anti-chondroitin sulfate (CS-56; Seikagaku Kogyo, Tokyo, Japan).

### Detection of endogenous SRPX2 protein

The culture medium was dialyzed against 50 mM of ammonium bicarbonate and lyophilized. The residue was dissolved in 50 mM of Tris-HCl (pH 7.4) and centrifuged at 20,000 rpm for 30 min. The supernatant was filtered through a 0.22-µm filter. The filtrate was subjected to fast protein liquid chromatography (FPLC; GE Healthcare UK Ltd. Buckinghamshire, England) separation on HiTrap Q HP columns (5 mL; GE Healthcare). The columns were equilibrated with 50 mM of Tris-HCl (pH 7.4). The samples were then injected onto the columns, which were washed with the same buffer and eluted at a flow rate of 4 mL/min using a linear gradient consisting of 0–2 M NaCl in 50 mM Tris-HCl (pH 7.4) over 45 min. The SRPX2 protein-containing fractions were then performed using gel-filtration chromatography (Superdex200 column, 16 mm×60 mm; GE Healthcare).

### Expression constructs and purification of SRPX2-HA/His protein

The method for producing the expression constructs was previously described [5]. Empty and SRPX2-HA/His vectors were then transfected into HEK293 cells using FuGENE6 transfection reagent (Roche Diagnostics, Basel, Switzerland), and the cells were then selected with hygromycin. The stable transfectant HEK293 cells were designated as HEK293-Mock and HEK293-SRPX2-HA/His. The conditioned medium of the HEK293-Mock and HEK293-SRPX2-HA/His cells was subjected to FPLC loading at 3 mL/min on a 5-mL HisTrap HP column (GE Healthcare). The bound protein was washed with 15 mL of wash buffer (WB: 50 mM Na_2_HPO_4_, 10 mM Tris-HCl, 20 mM imidazole [pH 8.0] and 600 mM NaCl,) and eluted in elution buffer (EB: WB+230 mM imidazole). The SRPX2-HA/His protein-containing fractions were applied to an FPLC Superdex200 column (16 mm×60 mm; GE Healthcare) equilibrated with 0.15 M of ammonium bicarbonate. Elution was carried out using the same buffer at a flow rate of 1 mL/min. The SRPX2-HA/His-containing fractions were verified using western blotting and lyophilized.

### Digestion of SRPX2 by specific GAG-degrading enzymes

Purified SRPX2-HA/His protein was digested with several specific enzymes including chondroitinase ABC and chondroitinase AC II (0.1 units in 40 mM Tris-HCl, 40 mM sodium acetate [pH 8.0] at 37°C for 2 h), chondroitinase B (0.02 units in 20 mM Tris-HCl, 0.25 µM calcium acetate [pH 7.5] at 37°C for 2 h), heparinase I and heparinase II (0.05 units in 5 mM calcium acetate, 50 mM sodium acetate [pH 7.0] 37°C for h), keratanase (0.1 units in 7.5 µM Tris-HCl [pH 7.4] at 37°C for 2 h), and hyaluroinidase (0.02 M acetate buffer, 0.15 M NaCl [pH 6.0] at 60°C for 2 h). Enzymes were purchased from Seikagaku Kogyo. The samples were then analyzed using western blotting.

### Binding Assays

An IAsys resonant mirror biosensor (Affinity Sensors, Cambridge, UK) with a carboxymethyl dextran-sensing cuvette was used to determine the kinetic constants of hepatocyte growth factor (HGF) binding to immobilized SRPX2-HA/His. SRPX2-HA/His was dissolved in 10 mM sodium formate (pH 4.0) and immobilized on the carboxymethyl dextran surface of the cuvette, according to the manufacturer's instructions. Binding experiments were performed in PBS. Changes in the resonant angle were monitored at 1-s intervals for approximately 600 s. Experiments were performed at 25°C with a stirrer speed of 80 rpm. The binding parameters were calculated from the association and dissociation phases of the binding reactions using the non-linear curve fitting FastFit (Affinity Sensors). Bovine serum albumin (BSA) was used as a control.

### Microarray data

The clinical samples of the paired colorectal cancers (CRCs), microarray procedure and analysis method have been previously described [7]. This study was approved by the institutional review board, and written informed consent was obtained from all the patients. All microarray data has been deposited to Center for Information Biology gene Expression database (CIBEX, http://cibex.nig.ac.jp/index.jsp) as accession number #CBX205. All data is MIAME compliant and that the raw data has been deposited in a MIAME compliant database (CIBEX), as detailed on the MGED Society website http://www.mged.org/Workgroups/MIAME/miame.html.

### Patients and samples

The 30 CRC and 10 paired non-cancerous colonic mucosa samples were analyzed using real-time RT-PCR. The RNA extraction method and the quality check protocol have been previously described [7]. This study was approved by the institutional review board of the National Cancer Center Hospital, and written informed consent was obtained from all the patients.

### Real-time reverse transcription PCR and western blot analysis

The methods used in this section have been previously described [5].

## Results

### Overexpression of SRPX2 in CRC tissues

**Figure 1:**
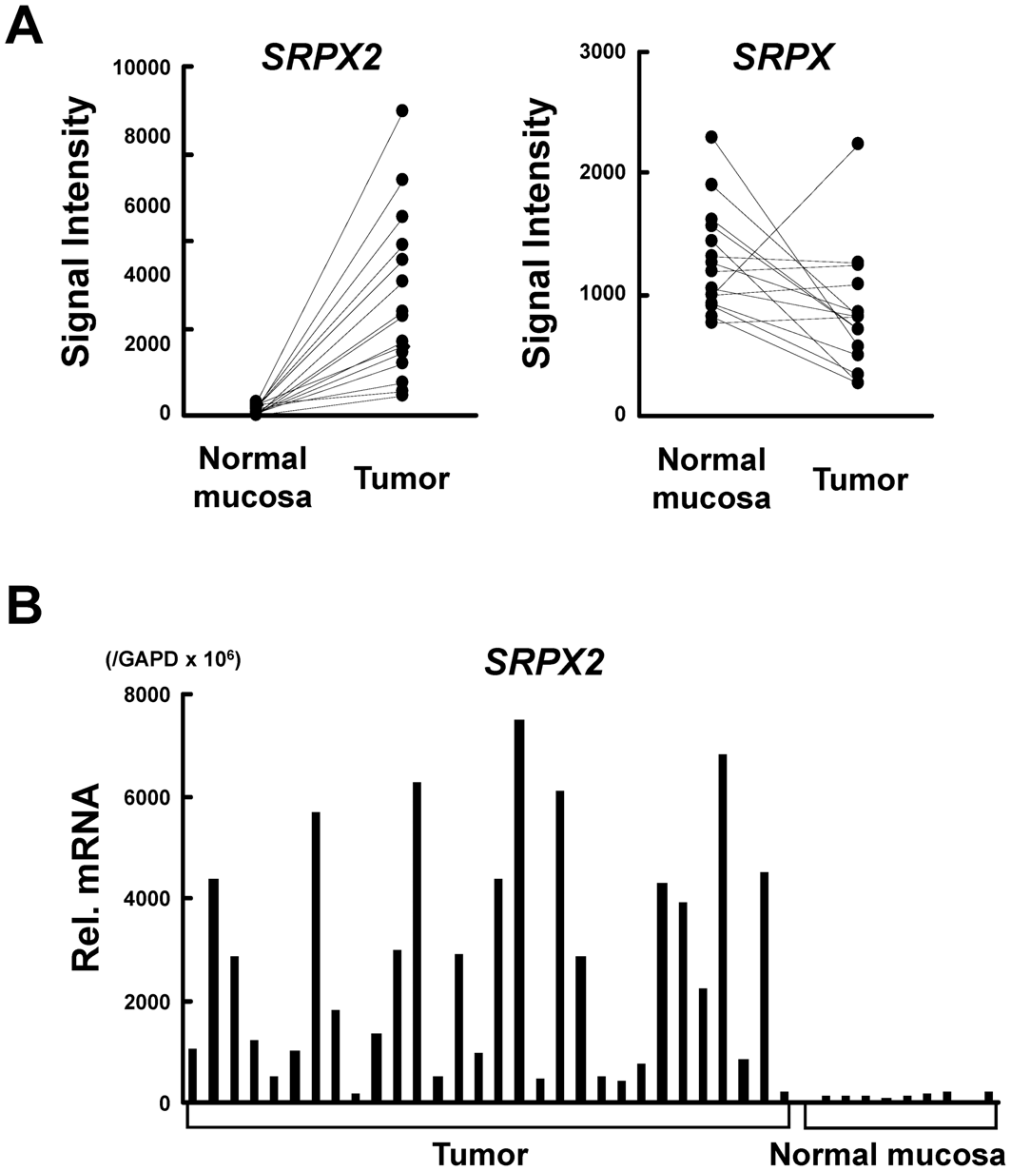
SRPX2 is overexpressed in colorectal cancer (CRC). (A) The mRNA expression of *SRPX2* and its homologue *SRPX* in 15 CRC and paired normal colonic mucosa specimens. The values indicate the normalized signal intensity obtained from the microarray data. (B) mRNA expression levels of *SRPX2* determined using real-time RT-PCR. CRC: colorectal cancer, Rel mRNA: normalized mRNA expression levels (*SRPX2*/*GAPD*×10^6^).

We evaluated the mRNA expression of *SRPX2* in clinical samples of CRCs in addition to its homologue *SRPX* (*SRPX1*) using microarray data. *SRPX2* expression was markedly up-regulated (20.5 fold, p = 0.00014) in cancer tissues, compared with paired noncancerous mucosa samples, whereas the putative tumor suppressor gene *SRPX* was down-regulated (0.7 fold, p = 0.029) in cancer (Fig. 1). The result indicates that *SRPX2* is overexpressed in CRC during carcinogenesis and tumor progression, unlike *SRPX*. Real-time RT-PCR for the 30 CRC and 10 paired non-cancerous colonic mucosa samples confirmed that *SRPX2* mRNA was markedly overexpressed in the CRC samples but was only expressed at a very low level in non-cancerous colonic mucosa (Figure 1B).

### Secreted SRPX2 protein is suspected to be modified posttranslationally

**Figure 2:**
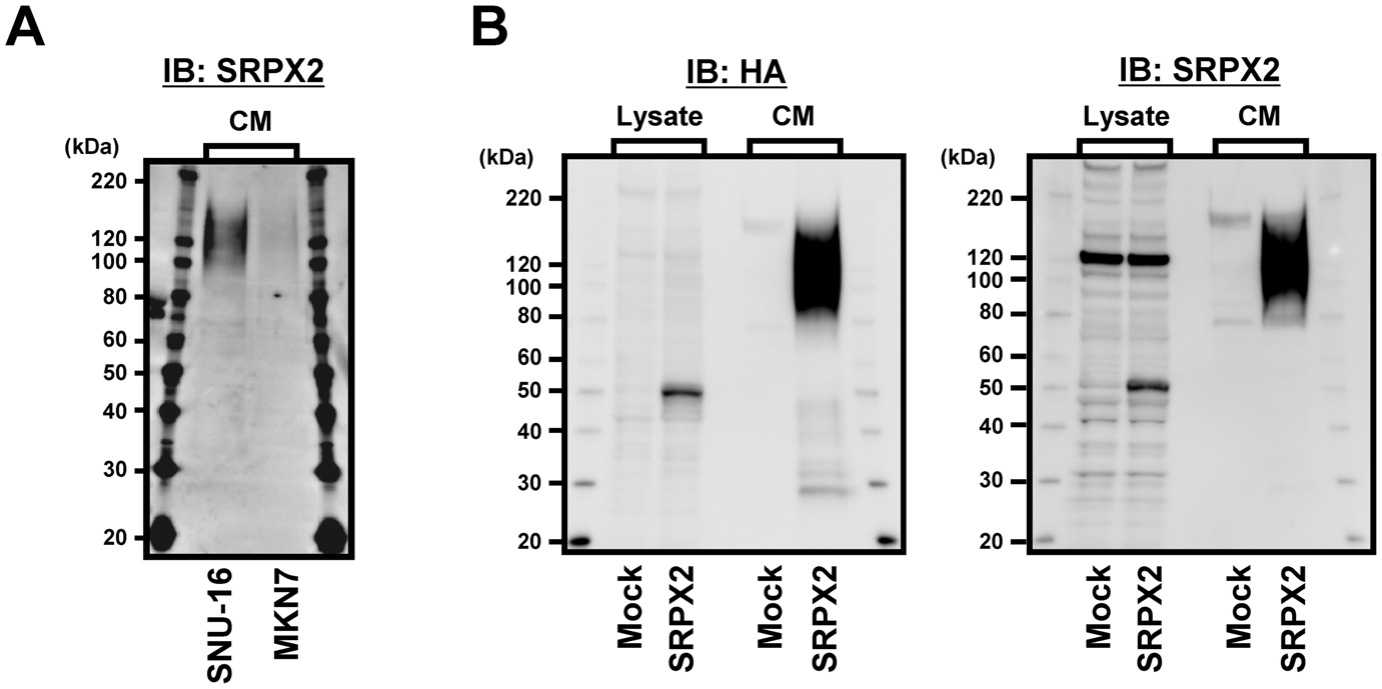
Secreted SRPX2 protein is suspected to be modified posttranslationally. (A) Secreted form of endogenous SRPX2 protein obtained from culture medium (CM) in SNU-16 and MKN7 cells. CM was subjected to ion exchange chromatography and used for western blotting analysis using anti-SRPX2 antibody. (B) Western blotting for exogenous SRPX2 protein obtained from cell lysate and CM using anti-SRPX2 and anti-HA antibody. Stable transfectant HEK293 cells, introducing the full-length cDNA fragment encoding human SRPX2 with HA and the His-tag vector or empty vector, were used for analysis. The non-smeared bands at 120 kDa in cell lysate are endogenous SRPX2. Mock: HEK293-Mock cells, SRPX2: HEK293-SRPX2-HA/His cells. IB: immunoblotting, Lysate: cell lysate, CM: culture medium.

The predicted molecular mass of SRPX2 protein was 53 kDa; however, western blotting revealed that the molecular mass of the secreted SRPX2 protein was highly increased, with smeared bands at an apparent molecular mass of 100–150 kDa in SNU-16 and MKN7 cell lines (Fig. 2A). Next, we evaluated the exogenously expressed SRPX2 protein derived from HEK293-Mock and HEK293-SRPX2-HA/His cells. The molecular mass of intracellular SRPX2 protein was similar to the predicted size, while the molecular mass of the secreted-SRPX2 protein was highly increased (100–150 kDa). Smeared bands were also detected using both anti-HA and anti-SRPX2 antibodies (Fig. 2B). The non-smeared bands at 120 kDa in cell lysate are endogenous SRPX2. These results suggested that secreted SRPX2 protein may undergo posttranslational modifications.

### SRPX2 is a novel chondroitin sulfate proteoglycan

**Figure 3:**
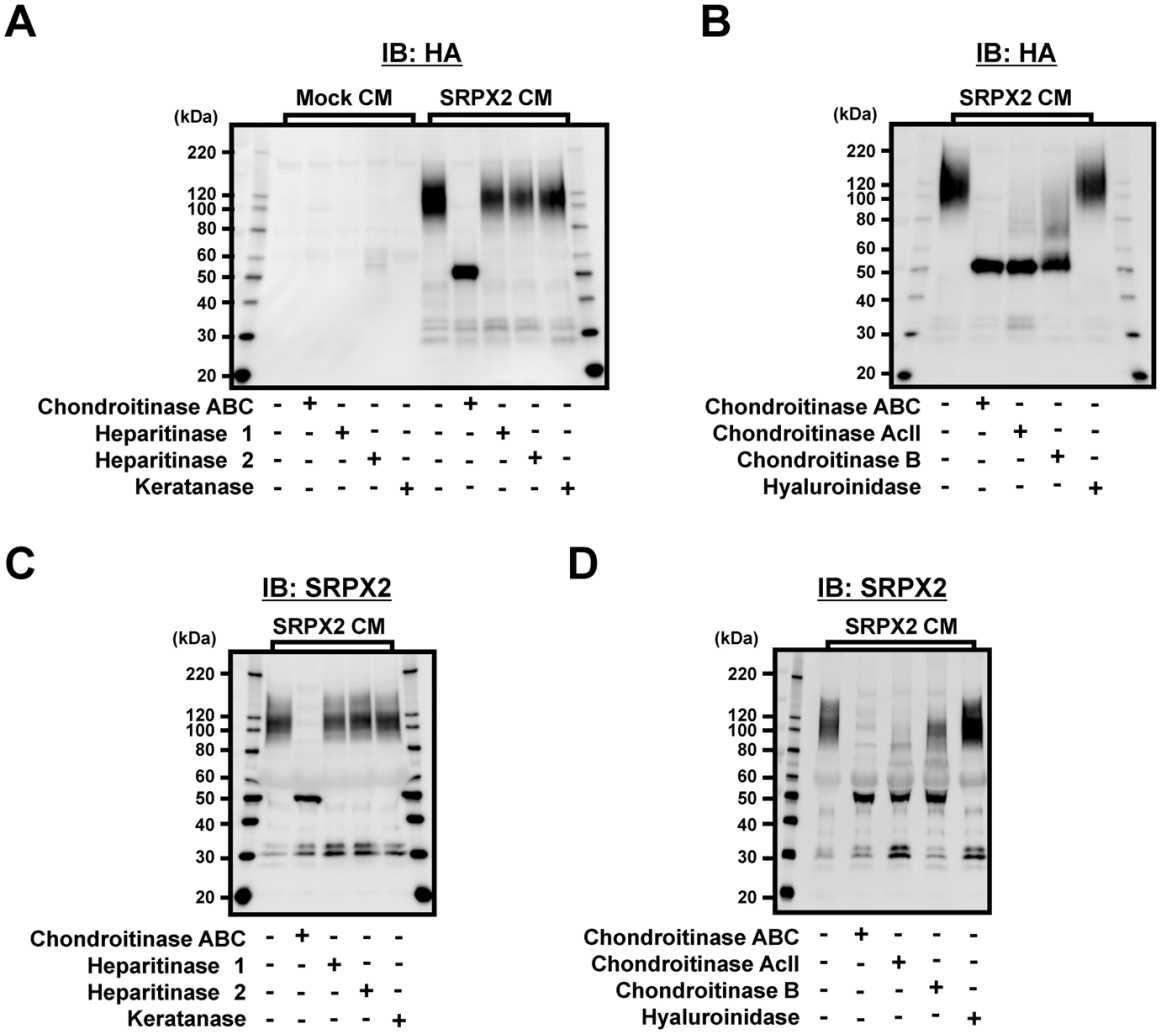
Effects of chondroitinases on SRPX2. (A, B) Purified SRPX2 protein obtained from cultured medium of HEK293-Mock or HEK293-SRPX2-HA/His cells were digested with chondroitinase ABC, heparitinase 1, heparitinase 2, keratanase, chondroitinase AcII, chondroitinase B and hyaluroinidase. The effect of digestion of the glycosaminoglycan chains was detected using western blotting using anti-HA (A, B) and anti-SRPX2 (C, D) antibody. IB: immunoblotting, CM: culture medium. Mock: HEK293-Mock cells, SRPX2: HEK293-SRPX2-HA/His cells.

**Figure 4:**
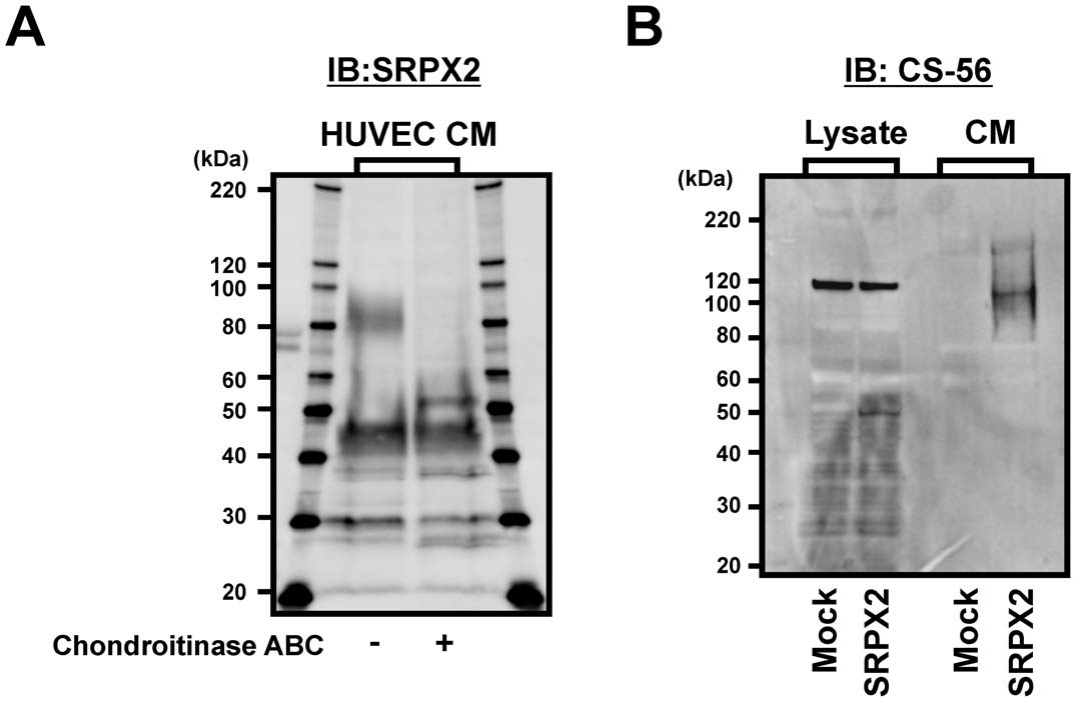
Detection of chondroitin sulfate glycosaminoglycan and binding of HGF to SRPX2. (A) Chondroitinase ABC digestion for endogenous SRPX2 protein derived from HUVEC (human umbilical vein endothelial cells). The SRPX2 protein was detected using anti-SRPX2 antibody. (B) Western blotting for SRPX2 protein using anti-chondroitin sulfate antibody (CS-56). The non-smeared bands at 120 kDa in cell lysate are endogenous SRPX2. IB: immunoblotting, Lysate: cell lysate, CM: culture medium. Mock: HEK293-Mock cells, SRPX2: HEK293-SRPX2-HA/His cells.

Based on the appearance of the smeared bands at a highly increased molecular mass, we hypothesized that SRPX2 is a proteoglycan with glycosaminoglycan (GAG) chains. Accordingly, we treated purified-SRPX2 protein obtained from the cultured medium of HEK293-Mock (empty control) or HEK293-SRPX2-HA/His cells with chondroitinase ABC, heparitinase 1, heparitinase 2, keratanase, chondroitinase AcII, chondroitinase B, and hyaluroinidase. Western blotting revealed that the molecular mass of the secreted SRPX2 protein was clearly decreased by chondroitinase ABC digestion, but not by heparitinase or keratanase or hyaluroinidase (Fig. 3A, 3B). Further chondroitinase treatment showed that chondroitinase ABC and chondroitinase AcII completely digested GAGs on SRPX2, but that chondroitinase B partially digested these chains (Fig. 3B). A small digested SRPX2 protein was also detected using anti-SRPX2 antibody (Fig. 3C, 3D). These results indicate that SRPX2 contains chondroitin sulfate GAG chains and is a novel chondroitin sulfate proteoglycan (CSPG). In addition, the partial digestion by chondroitinase B suggests that a dermatan sulfate component may be included in the chondroitin sulfate GAG chains. Next, we confirmed the results of enzymatic digestion against endogenous SRPX2 from HUVEC using western blotting with anti-SRPX2 antibody and a similar result was obtained (Fig. 4A). Anti-chondroitin sulfate antibody (CS-56) also detected the chondroitin sulfate GAG on SRPX2 (Fig. 4B). The non-smeared bands at 120 kDa in cell lysate are endogenous SRPX2.

### HGF binds to SRPX2

**Figure 5:**
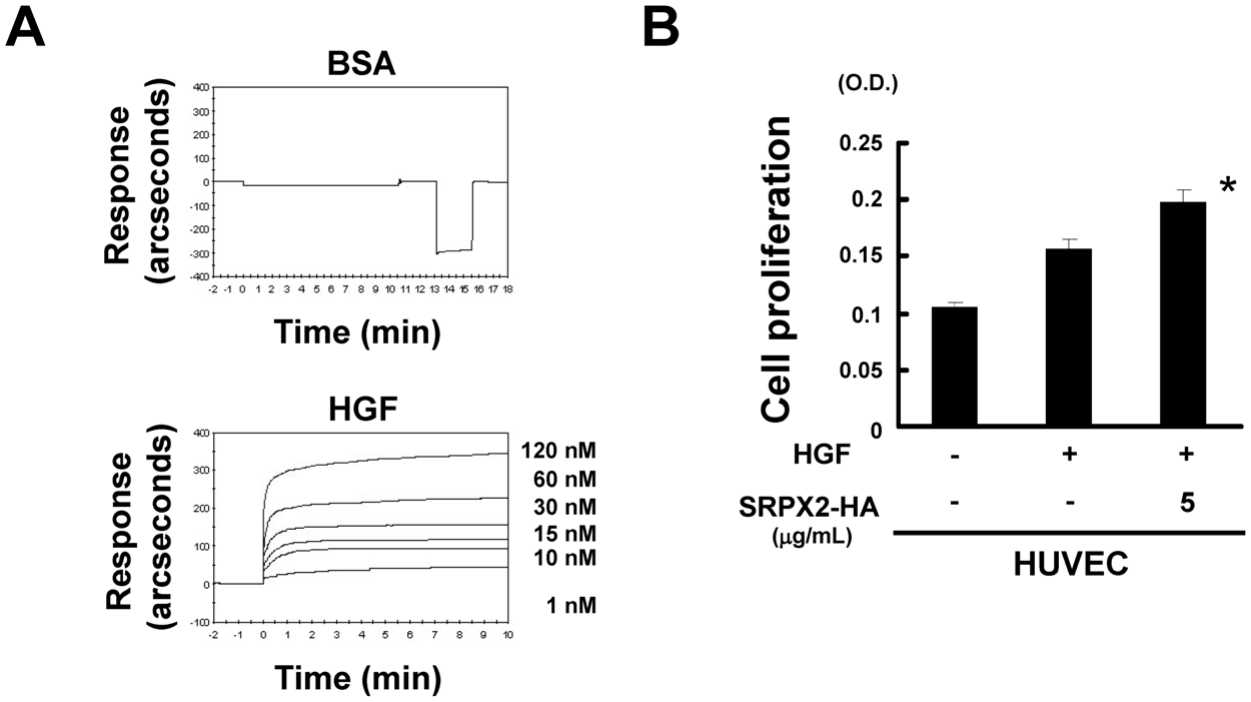
Binding of HGF to SRPX2 at the indicated concentrations. (A) IAsys resonant mirror biosensor was used for analysis. Bovine serum albumin (BSA) was used as a negative control. (B) Cell proliferation of HUVECs evaluated using an MTT assay. The HUVECs were stimulated with or without 10 ng/mL of HGF and 5 µg/mL of purified SRPX2 protein for 72 hours. *, SRPX2 (−) vs. (+), p<0.05.

It is well known that several ligands including HGF, heparin-binding EGF-like growth factor, fibroblast growth factor 2 and vascular endothelial growth factor are capable of binding to the GAG chain and that such interactions are considered to be a unique characteristic of GAGs and proteoglycans [8]. According to a report on CSPG endocan and HGF binding [9], we examined the interaction between HGF and GAGs using an IAsys resonant mirror biosensor. HGF dose-dependently bound to the GAGs of SRPX2, while control BSA did not (Fig. 5A). The *K_d_* value of this interaction, calculated from the ratio of *K_diss_/K_ass_*, was 5.6 nM; these data were similar to those for previously reported data on HGF and endocan [9]. Next, we examined the biological function of SRPX2 on HGF. HGF increased the proliferation of HUVECs, and the addition of purified SRPX2 protein into the medium significantly increased HGF-induced proliferation (Figure 5B). These results suggest that the interaction of HGF with SRPX2 has a positive effect on angiogenesis.

### SRPX2 has unique molecular architectures compared with other sushi repeat module-containing CSPG

**Figure 6:**
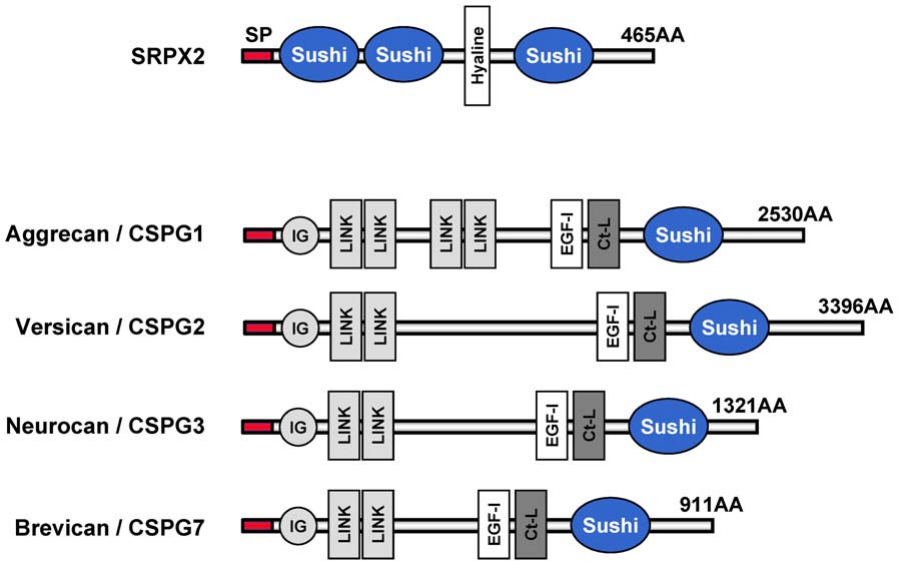
Molecular architectures of SRPX2. The data was obtained from the public database SMART (http://smart.embl-heidelberg.de/). SRPX2 has three sushi repeat modules and one hyaline domain. Four sushi repeat module-containing CSPG (agrrecan, versican, neurocan and brevican; also known as lecticans) are also shown. SP: signal peptides, AA: amino acids. Sushi: sushi repeat modules/CCP/short consensus repeats, Hyaline: hyaline domain, IG: immunoglobulin-like, LINK: hyaluronan-binding, EGF-l: EGF-like (Ca^2+^-binding), Ct-L: C-type lectin.

Data from publicly available databases (http://smart.embl-heidelberg.de/) and a previous report [10] showed that SRPX2 has three sushi repeat modules (also known as complement control protein modules or short consensus repeats) and one hyaline domain (Fig. 6). Interestingly, four CSPG (agrrecan, versican, neurocan and brevican; also known as lecticans) are present among the sushi repeat module-containing family, and their common molecular architectures consist of one immunoglobulin-like domain, 2∼4 LINK domains, one EGF-like domain, one C-type lectin, and one sushi repeat module (Fig. 6). The presence of a sushi repeat module and classification as a CSPG are the same for SRPX2 and lecticans, but the other molecular architectures of SRPX2 are quite different.

Taken together, these findings indicate that SRPX2 is a novel CSPG that is overexpressed in gastrointestinal cancer cells.

## Discussion

The extensive use and structural diversity of sushi repeat modules presumably reflects the versatility of a structural scaffold that has been adapted by evolution to suit many purposes, both architectural and functional, such as the mediation of specific protein-protein and protein-carbohydrate interactions [10]–[12]. Meanwhile, SRPX2 has one hyaline domain, which appears to be involved in cellular adhesion. Hyaline domains have been identified in several eukaryotic proteins and are often associated with sushi repeat modules or arranged in multiple copies [13]. These characteristics of the molecular architectures of SRPX2, based on knowledge of protein-protein interactions, may contribute to ligand/receptor interactions between SRPX2 and uPAR, with implications for disorders of the language cortex, cognition, and angiogenesis [3], [4].

We have demonstrated that SRPX2 is a novel CSPG, suggesting that SRPX2 may have additional as yet unknown biological functions as a proteoglycan, including interactions with various extracellular signaling molecules such as growth factors, morphogens, enzymes and chemokines and/or may act at the cell-extracellular-matrix interface to modulate cell signaling. The conditioned-medium of SRPX2-producing cells markedly enhanced cellular adhesion in various cancer cell lines [5]; this result can be explained by the biological function of SRPX2 as a proteoglycan. In addition, although we have only demonstrated that HGF can bind to SRPX2, our results suggest that other known GAG-interacting ligands may be capable of binding to the GAG chain of SRPX2. Therefore, the function of ligand-SRPX2 binding may widely affect the activities of signaling pathway critical to cancer cells, including cellular proliferation, apoptosis, migration and survival [14]. In addition, SRPX2 was found to be secreted and may act as an extracellular matrix protein similar to other proteoglycans; indeed coating the culture dish with SRPX2 protein markedly enhanced cellular adhesion [5], supporting this idea.

Vascular endothelial cells HUVEC markedly express SRPX2 to the same extent as high-expressing cancer cell lines [5]. A recent report demonstrated that Srpx2 is a novel mediator of angiogenesis and a key molecule involved in the invasive migration of angiogenic endothelium through its role as a ligand for vascular uPAR [4]. Our findings also support the involvement of SRPX2 in angiogenesis from another aspect of proteoglycans. Since endocan is well-known as a vascular endothelial cells-specific CSPG [8], SRPX2 may be categorized as a vascular-related CSPG similar to endocan.

In conclusion, we found that SRPX2 is a novel chondroitin sulfate proteoglycan that is overexpressed in gastrointestinal cancer. Our findings provide key glycobiological knowledge of this protein in cancer cells.
